# Supplementary material for: Deciphering the Potential Coding of Human Cytomegalovirus: New Predicted Transmembrane Proteome
Source: Int J Mol Sci. 2022 Mar 2;23(5):2768. doi: 10.3390/ijms23052768 (PMC8911422; doi:10.3390/ijms23052768)
Supplement: Supplementary file 1 [file ijms-23-02768-s001.zip › Supplementary captions.pdf]

Supplementary Materials

Figure S1. Representative analysis of the selected nine strains. (A) The 56,217 ORFs of the 335 CMV genome sequences available in the database were aligned using Blastp with the ORFs included in our dataset (9 representative genomes). Percentage and identity for all hits indicate a higher distribution of approximately 100% in both cases. (B) Violin representation. The 335 CMV genome sequences were aligned with the ORFs coding for the 39 core proteins using blastp under default parameters. A high number of genomes contained all 39 core proteins.

Figure S2. Predicted transmembrane proteins for the studied CMV genomes. We have included the information of a group of 17 proteins predicted to have transmembrane regions (ranging from 1–3) that were discarded for further analysis because mainly they were known to have a function that was not related with membrane location. The number of transmembrane regions for each protein was represented using a chromatic scale ranging from zero to eight regions in total in the three applied methods (PureseqTM, Phobius and TMHMM). For each strain, the presence of the gene was represented with the filled red circles and absent genes with empty red circles.

Figure S3. Western Blot validation of the predicted transmembrane proteins UL4 and UL124. Ten micrograms of protein lysates from mock HEK 293T cells (Lane 1 and 2) and HEK 293T transfected cells with UL4 (Lane 3 and 4) and UL124 (Lane 5 and 6) were used. As the primary antibody, an anti Myc antibody was used. Stain free was used as loading control. C: Cytoplasmic fraction and PM: Plasma membrane fraction. Figure S4. Similarity matrix. Percentage identity heatmaps for the 39 core transmembrane proteins among the nine studied CMV strains. Color ranges from red to white (0% and 100% of identity, respectively).

Table S1. Analysis results of the complete transmembrane (Phobius, PureseqTM and TMHMM) and functionality (Mantis) workflow for all CMV analyzed strains. ACC: accession number and gene name; info: protein name and accession number; Start: gene starting nucleotide position in the genome sequence; End: gene ending nucleotide position in the genome sequence; Strand: directionality of the gene; pureseqtm_ntrans: number of transmembrane sequences detected by PureseqTM; pureseqtm: transmembrane nucleotide position in the genome sequence using PureseqTM; phobius_ntrans: number of transmembrane sequences detected by Phobius; phobius: transmembrane nucleotide position in the genome sequence using Phobius; tmhmm_ntrans: number of transmembrane sequences detected by TMHMM; tmhmm: transmembrane nucleotide position in the genome sequence using TMHMM; Function: most related function found following Mantis workflow; Function from Orthologues: most related function found from related orthologues sequences; SP:Signal peptide.

Table S2. List of oligonucleotides used in this study.
