# Supplementary material for: Deciphering the Potential Coding of Human Cytomegalovirus: New Predicted Transmembrane Proteome
Source: Int J Mol Sci. 2022 Mar 2;23(5):2768. doi: 10.3390/ijms23052768 (PMC8911422; doi:10.3390/ijms23052768)
Supplement: Supplementary file 1 [file ijms-23-02768-s001.zip › Table S2.pdf]

Table S2. Oligonucleotides used in this study for amplification and sequencing of validated CMV proteins.

Oligonucleotide	Sequence (5'→3')
UL2-XhoI-F	CCGCTCGAGCGGCCACCATGGCCGAAGACTCGGTCGC
UL2-KpnI-R	CGGGGTACCCCGATAAAAGAGCGTCTCGAAGCA
UL124-EcoRI-F	CCGCTCGAGCGGCCACCATGGAAAGGAACAGTCTGTT
UL124-KpnI-R	CGGGGTACCCCGAAACATAACGTGGGATCTCCA
pcDNA-F-seq	GTAGGCGTGTACGGTGGGAG
pcDNA-R-seq	CAGTCGAGGCTGATCAGCGG

The recognition site of the enzyme is marked in red indicating the exact cut site. The oligonucleotides pcDNA-F-seq and pcDNA-R-seq were used to sequence all the tested genes because they are specific to up- and down-stream sequences of the multiple cloning site of the plasmid.
