# Supplementary figures and images for: Deciphering the Potential Coding of Human Cytomegalovirus: New Predicted Transmembrane Proteome
Source: Int J Mol Sci. 2022 Mar 2;23(5):2768. doi: 10.3390/ijms23052768 (PMC8911422; doi:10.3390/ijms23052768)

**A**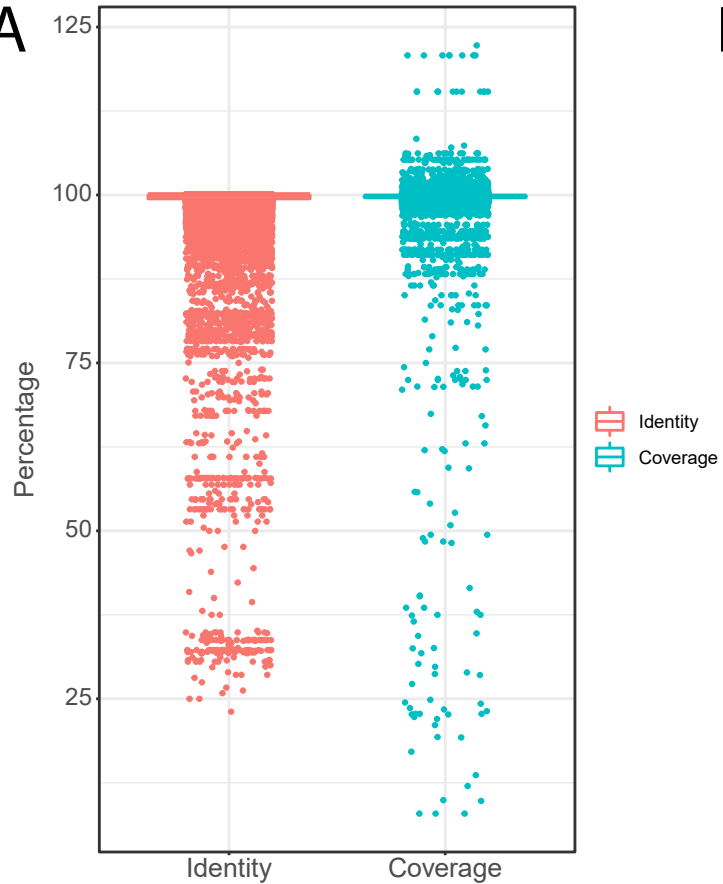**B**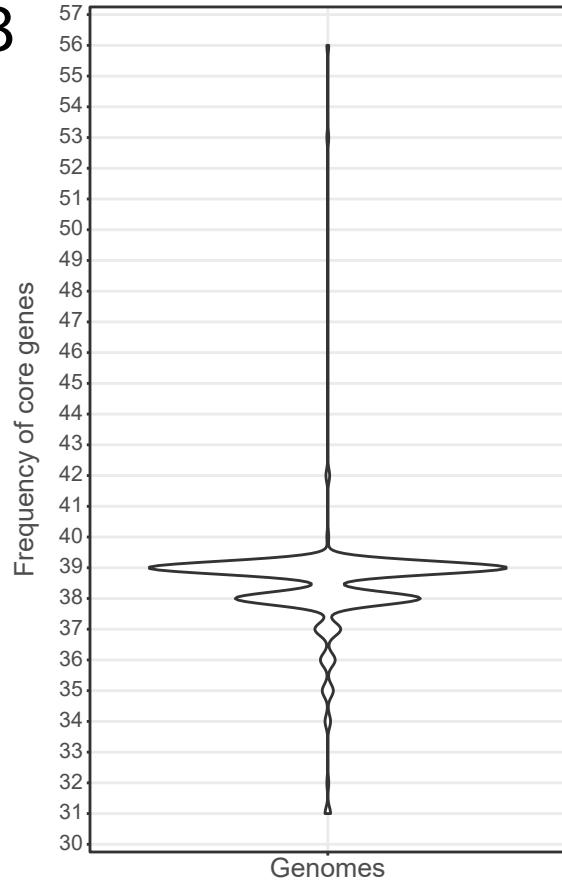

Supplement: Supplementary file 1 [file ijms-23-02768-s001.zip › Figure S1.pdf]

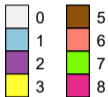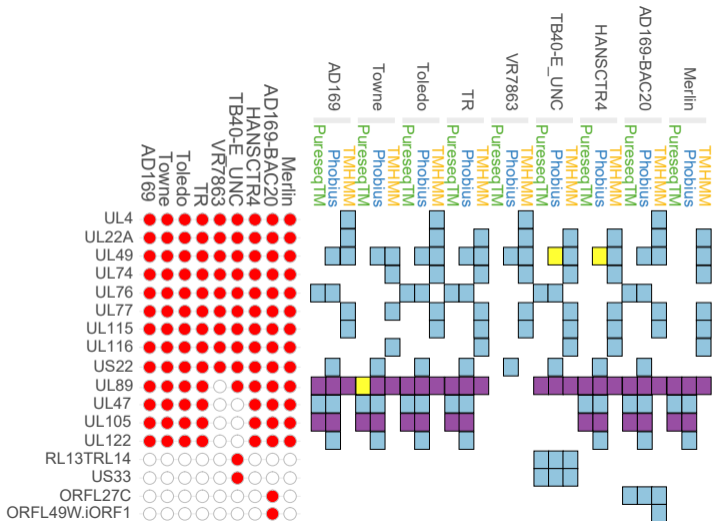

Supplement: Supplementary file 1 [file ijms-23-02768-s001.zip › Figure S2.pdf]

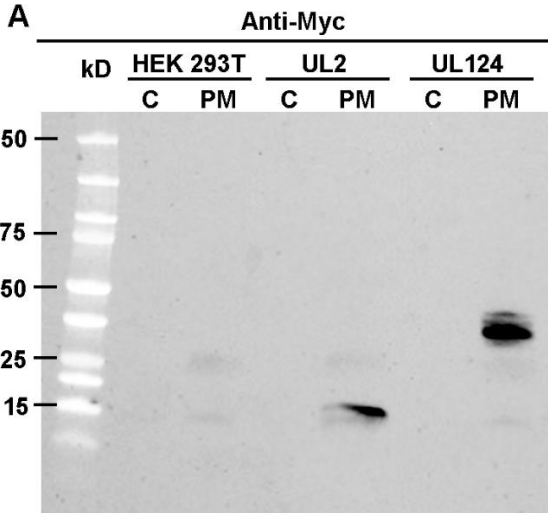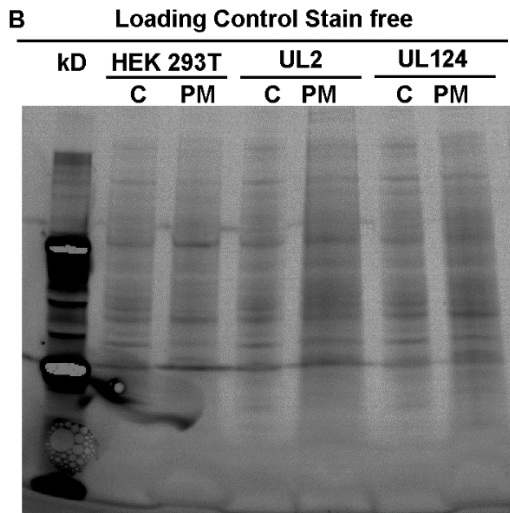

Supplement: Supplementary file 1 [file ijms-23-02768-s001.zip › Figure S3.pdf]

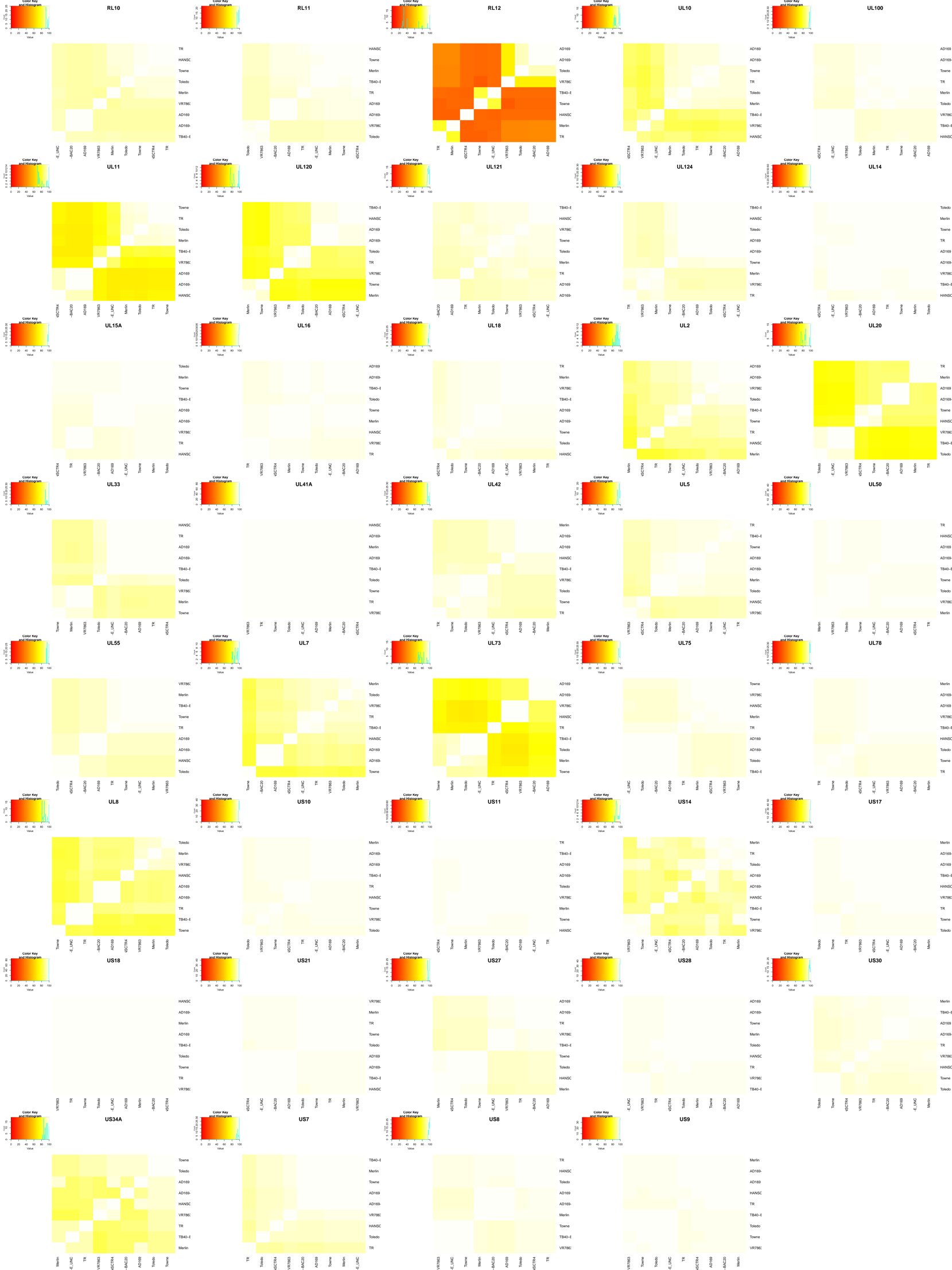

Supplement: Supplementary file 1 [file ijms-23-02768-s001.zip › Figure S4.pdf]
